# Self-aligning prosthetic device for older patients with vascular-related amputations: protocol for a randomised feasibility study (the STEPFORWARD study)

**DOI:** 10.1136/bmjopen-2019-032924

**Published:** 2019-09-20

**Authors:** Natasha Mitchell, Elizabeth Coleman, Judith Watson, Kerry Bell, Catriona McDaid, Cleveland Barnett, Martin Twiste, Fergus Jepson, Abayomi Salawu, Dennis Harrison, Natalie Vanicek

**Affiliations:** 1 York Trials Unit, Department of Health Sciences, University of York, York, UK; 2 School of Science and Technology, Nottingham Trent University, Nottingham, UK; 3 United National Institute for Prosthetics & Orthotics Development, University of Salford, Salford, UK; 4 Specialist Mobility Rehabilitation Centre, Lancashire Teaching Hospitals NHS Foundation Trust, Preston, UK; 5 Disability Medicine and Rehabilitation Unit, Hull University Teaching Hospitals NHS Trust, Hull, UK; 6 Public involvemment member, Hull, UK; 7 Department of Sport, Health and Exercise Science, University of Hull, Hull, UK

**Keywords:** Randomised controlled trial, feasibility, amputation, prosthesis, acceptability, vascular

## Abstract

**Introduction:**

The majority of older patients with a transtibial amputation are prescribed a standard (more rigid, not self-aligning) prosthesis. These are mostly suitable for level walking, and cannot adjust to different sloped surfaces. This makes walking more difficult and less energy efficient, possibly leading to longer term disuse. A Cochrane Review concluded that there was insufficient evidence to recommend any individual type of prosthetic ankle-foot mechanism. This trial will establish the feasibility of conducting a large-scale trial to assess the effectiveness and cost-effectiveness of a self-aligning prosthesis for older patients with vascular-related amputations and other health issues compared with a standard prosthesis.

**Methods and analysis:**

This feasibility trial is a pragmatic, parallel group, randomised controlled trial (RCT) comparing standard treatment with a more rigid prosthesis versus a self-aligning prosthesis. The target sample size is 90 patients, who are aged 50 years and over, and have a transtibial amputation, where amputation aetiology is mostly vascular-related or non-traumatic. Feasibility will be measured by consent and retention rates, a plausible future sample size over a 24-month recruitment period and completeness of outcome measures. Qualitative interviews will be carried out with trial participants to explore issues around study processes and acceptability of the intervention. Focus groups with staff at prosthetics centres will explore barriers to successful delivery of the trial. Findings from the qualitative work will be integrated with the feasibility trial outcomes in order to inform the design of a full-scale RCT.

**Ethics and dissemination:**

Ethical approval was granted by Yorkshire and the Humber—Leeds West Research Ethics Committee on 4 May 2018. The findings will be disseminated via peer-reviewed research publications, articles in relevant newsletters, presentations at relevant conferences and the patient advisory group.

**Trial registration number:**

ISRCTN15043643.

**Strengths and limitations of this study**

- This will be one of the few studies involving older amputees in research.
- Should it be determined that a larger scale randomised controlled trail is feasible, the information collected in this study will be vital in influencing the design.
- The proposed sample size is large enough to allow for reliable estimates to be obtained for calculating a future sample size for a main trial.
- The study is not large enough to determine effectiveness and is limited to assess the feasibility aspect.

## Introduction

The number of people with a lower limb amputation is growing and is predicted to double in developed nations by the year 2050.^1^ In the UK, there are approximately 6000 new referrals to prosthetics services every year, most commonly at the transtibial level.^2 3^ Most lower limb amputations (60%–80%) are related to ischaemia, vascular atherosclerotic disease and diabetic complications, and typically occur in people over 50 years of age.^3 4^ A retrospective review of hospital data in England reported that there were 25 312 lower limb amputations for patients aged between 50 and 84 years between 1 April 2003 and 31 March 2009.^5^ Therefore, most new referrals for a prosthesis involve older patients who usually present with multiple health comorbidities.

Prosthetic prescription, one important factor in the long-term outcomes following amputation, is multifaceted, and influenced by factors such as estimation of patient outcomes, patient goals and budget.^6^ Practice varies across UK prosthetics centres and is frequently cost-driven. In December 2016, NHS England issued its Clinical Commissioning Policy guidelines for the routine prescription of microprocessor-controlled prosthetic knees for people with a transfemoral amputation.^7^ The recent clinical commissioning policy is ground-breaking for people with a transfemoral amputation, but presents no advantage for the prescription of a prosthesis for individuals with an amputation at the transtibial level. A Cochrane Review concluded that there was insufficient evidence from ‘high-quality comparative studies for the overall superiority of any individual type of prosthetic ankle-foot mechanism’ in patients with a lower limb amputation.^8^ Subsequently, the majority of older people with a transtibial amputation, often due to vascular reasons, are prescribed a standard prosthesis, such as the non-articulated solid ankle cushioned heel (SACH), uniaxial or multiaxial prosthetic foot. These prostheses are unable to adjust to the different walking surfaces people encounter daily (ie, uneven terrain, slopes and stairs). This makes walking more difficult, less energy efficient and more tiring, and may lead to longer term disuse.

Lower limb amputation, and its associated health comorbidities (ie, disease status, age-related and amputation-related complications, pain and sedentary behaviour), present a burden for patients (and their carers). Patients often report having problems related to mobility, which subsequently impacts negatively on their independence, and socioeconomic well-being,^9 10^ especially among older, vascular amputees.^11^ Identifying a suitably functional prosthesis could bring many benefits to a patient’s life following amputation.

People with a lower limb amputation are an under-represented group of patients, with little research being carried out involving those aged over 50 years. We carried out a number of public involvement sessions in order to refine our study aims and identify elements of particular resonance with older individuals following their amputation. Public involvement members, who experienced reduced mobility, identified their standard prosthesis as a limiting factor in their everyday function. A poorly functioning prosthesis often contributed to sedentary behaviour, pain, more frequent visits to healthcare services, disuse of the prosthesis and possible isolation and overall poor quality-of-life.

In order to clearly identify an amputee’s current and potential functional status the insurance group, Medicare, established Medicare Functional Classification Levels (also called K levels) in 1995, which is widely used internationally. It is a method of quantifying need and the potential benefit of prosthetic devices for those with a lower limb amputation. Five classification levels (K0: low functional level to K4: high functional level) were established.^12^ An alternative classification system is the SIGAM (Special Interest Group in Amputee Medicine) developed by the British Society of Rehabilitation Medicine which measures levels of mobility (grade A: lowest mobility to grade F: highest mobility).^13^ A self-aligning prosthesis has been designed specifically for the K2 user, categorised as ‘limited mobility’, to alleviate many of the patient-reported limitations of a standard prosthesis. This prosthesis adjusts to slopes and steps via hydraulic mechanisms. A self-aligning prosthesis can improve ground clearance to avoid a fall, and aligns to secure the biological knee in individuals with a transtibial amputation, which is important for falls prevention.^14^ Based on laboratory studies, this type of self-aligning prosthesis also has demonstrated reduced residuum-socket interface pressures,^15^ which could alleviate pain in the residuum longer term. Although a self-aligning prosthesis is more expensive than a standard prosthesis (by approximately £800), the potential patient benefits through increased mobility, quality-of-life and fewer falls could offset future healthcare and socioeconomic costs. Previous studies have shown that more functional prostheses may offer better patient function and improve mobility in people with a lower limb amputation.^16 17^ However, these studies have not been undertaken in older patients and therefore have not considered the health comorbidities that affect older, often vascular, patients compared with younger, more active amputees.

## Methods and analysis

### Aim and objectives

The primary aim of this study is to determine the feasibility of conducting a full-scale randomised controlled trial (RCT) of the effectiveness and cost-effectiveness of a self-aligning prosthesis for older patients with vascular-related amputations and other health issues compared with a standard prosthesis.

The specific objectives are to:

- Determine patient recruitment rates.
- Explore any barriers to recruitment and how these might be overcome from the perspective of patients.
- Identify the most important outcomes to the patients.
- Assess the acceptability of the study procedures to both participants and recruiting centres.
- Measure patient use of NHS resources over the study period.
- Identify a primary outcome measure(s) for a future main trial.
- Assess the completeness of follow-up data, to establish how feasible it is to collect patient-reported outcome measures.
- Measure day-to-day use of the prosthesis in both groups and measure normal physical activity through the use of wearable technologies (activity monitors).

In addition, the feasibility of a future trial will be assessed based on whether:

- Study consent/retention rates and proposed sample sizes indicate recruitment for the full-scale RCT is plausible within a 24-month period.
- Outcome measures and fidelity evaluation data are successfully collected.
- There are no significant barriers to delivery of the trial, identified by participants or recruiting centres, that cannot be overcome.

### Design

The STEPFORWARD trial is a multicentre, mixed-methods, randomised controlled, open feasibility trial to assess the possibility of conducting a full-scale RCT of a self-aligning prosthesis for older patients with vascular-related amputations and other health issues compared with a standard prosthesis. The 2-year study commenced on 1 April 2018; participant recruitment started in July 2018.

### Settings and participants

Participants who meet the eligibility criteria are being recruited from multiple centres based in NHS Trusts across England.

### Inclusion criteria

A patient is deemed eligible if they meet all the following criteria:

1. Aged 50 years or over.
2. Has a unilateral amputation.
3. Has a transtibial amputation only.
4. Has an amputation due to vascular reasons (eg, diabetes, peripheral vascular disease), neurological disorders (eg, diabetic neuropathy) or life-limiting illness (eg, tumour, cancer).
5. Is categorised as ‘limited mobility’: K2 classification, or SIGAM mobility grade C or D.
6. Is currently using a standard prosthetic ankle-foot (eg, SACH, uniaxial, multiaxial (eg, multiflex) or other K1/K2 feet) that does not adjust to sloped surfaces and is not self-aligning.
7. Has been using a prosthesis for at least 12 months, with the same socket for a minimum of 3 months.
8. Has had a stable residual limb for at least 3 months (ie, stable in volume and without cuts or wounds; daily management of volume with socks and liners is acceptable).
9. Is willing to trial a new prosthesis for a 12-week period (if allocated to intervention arm).
10. Is able to self-complete the English language outcome measure tools (or complete with assistance).
11. Is able to follow the detailed verbal instructions required for the functional/clinical tests.
12. Is able to provide written informed consent.

### Exclusion criteria

Patients will be considered ineligible if any of the following apply:

1. Has contraindications of wearing their current prosthesis (eg, open wound, infection).
2. Has contraindications of wearing the novel prosthesis according to manufacturer’s instructions (eg, body mass ≥150 kg; build height (ie, distance between distal end of socket and ground) <115 mm).
3. Has had a recent cerebrovascular event, such as a stroke.
4. Has a disease that severely affects their memory, such as dementia or Alzheimer’s.

### Sample size

This research is a feasibility RCT and therefore does not have a primary outcome measure to inform a power calculation. Sample sizes of between 24 and 70 have been recommended for feasibility trials to allow for the reliable estimation of a SD for use in future sample size calculations.^18 19^ We plan to recruit a total of 90 patients in this study over a 16-month timeframe. Allowing for a 20% attrition rate we intend to have 72 patients in the final analysis.

### Randomisation

Following the completion of all baseline measures, participants will be randomised into one of two trial arms: standard prosthesis (standard treatment) or self-aligning prosthesis (novel treatment). Randomisation will be performed by the York Trials Unit. Participants will be individually randomised and stratified according to prosthetics centre on a 1:1 basis.

### Blinding

By the nature of the interventions used within this study, blinding of the participants and investigators is not possible and procedures for breaking codes/unblinding are not necessary.

### Intervention and standard treatment

#### Novel treatment

The self-aligning ankle-foot prosthesis is called the Avalon-K2, manufactured by Blatchford and Sons, UK (Patent reg: 5336386). The prosthesis is already commercially available and may be prescribed under the NHS.

Following randomisation, participants in the novel treatment group will have an initial meeting with their regular prosthetist for fitting of the novel prosthesis. Once fitted with the self-aligning prosthesis, participants will be asked to acclimatise and ambulate with it as they would normally with any new prosthesis for approximately 12 weeks after fitting (intervention period). They will be offered physiotherapy sessions, to ensure their ability to ambulate safely with the new prosthesis, based on clinical need.

Participants in the novel treatment group will be given the option to keep using the novel prosthesis after the trial has completed or return to their original prosthesis.

#### Standard treatment

Participants in the standard treatment group will continue using their normal ankle-foot prosthesis. Participants in this group will continue to receive standard treatment and have access to all clinical services as normal. Standard treatment usually consists of routine visits to the consultant and/or patient-initiated visits (ie, normal prosthetics maintenance, and/or trouble-shooting due to prosthesis malfunction). This group will be asked to go about their normal daily routine wearing their standard prosthesis for 15 weeks (intervention period plus additional 3 weeks to allow for ordering and fitting of new prosthesis for participants in the novel group).

Participants in the standard treatment group will not be offered to sample the self-aligning prosthesis at the end of the trial as it is not known whether the novel prosthesis is better and more acceptable to patients than the standard prosthesis.

### Participant identification and recruitment process

**Figure 1:**
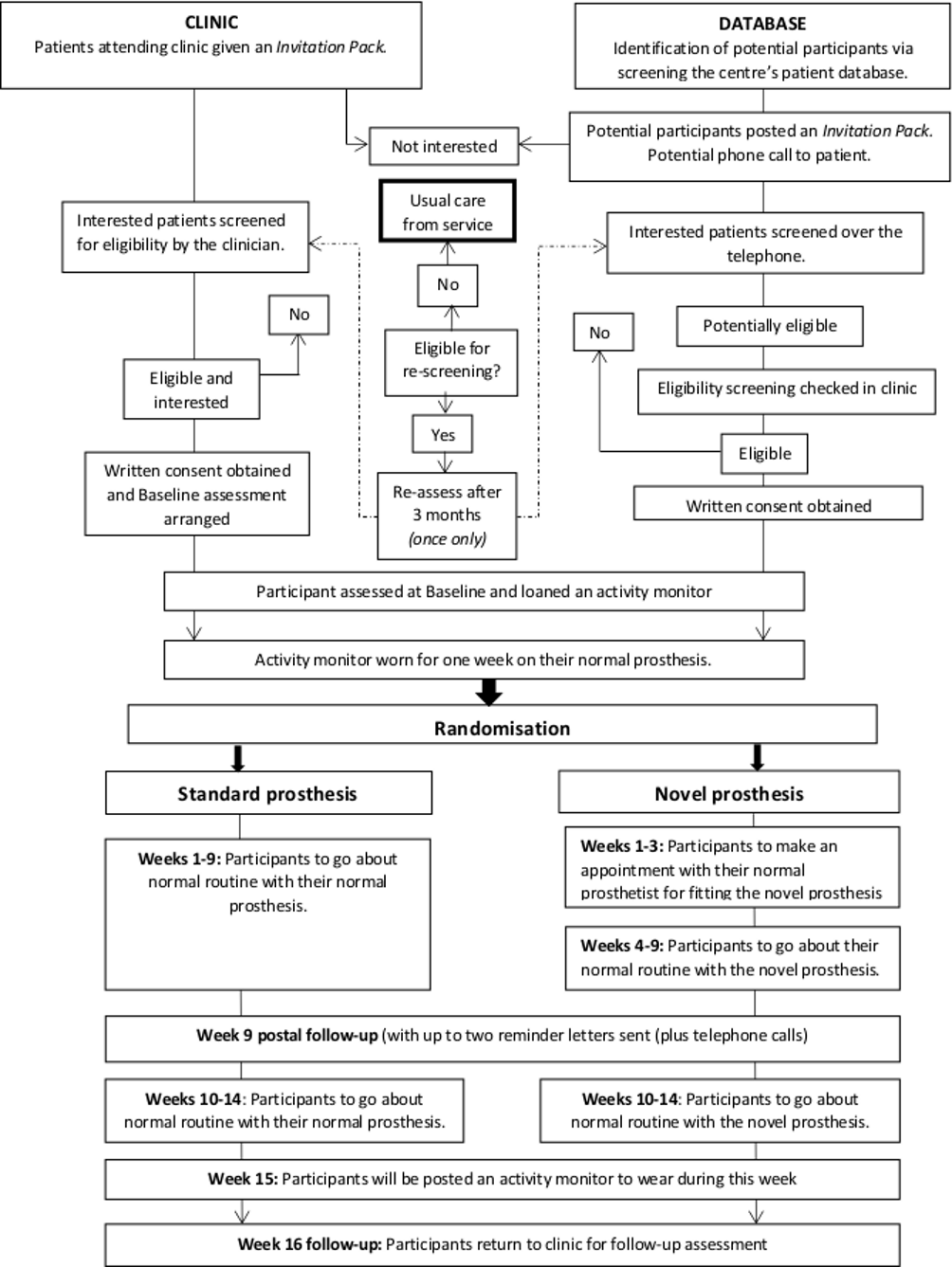
The STEPFORWARD study flowchart.

All patients with a unilateral, transtibial amputation due to non-traumatic reasons will be screened for eligibility. The practicalities of using the eligibility criteria will be assessed to inform the criteria that may be subsequently used in a main trial. Prospective participants will be identified via two main methods: during a routine clinical visit or via screening of the patient database (figure 1).

#### In clinic

Potentially eligible patients attending for their routine appointment to the prosthetics centre will be approached and given a study invitation pack. Patients may complete a Consent to Contact form during their routine appointment, and be screened by a member of their multidisciplinary team (MDT) as soon as possible thereafter. Alternatively, patients may take the pack away to read through, and return their Consent to Contact form in the post. Then, the enrolment process will follow the one outlined for those identified via database screening.

#### Database screening

A member of the patient’s routine MDT and/or prosthetics centre manager will screen the prosthetics centre’s database for suitable patients according to the inclusion criteria. Potential participants will be posted a study invitation pack. They will be asked to read through the documents and return the Consent to Contact form if they wish to be contacted about the trial. If no Consent to Contact form is received, patients will be contacted by the prosthetics centre approximately 2 weeks after expected receipt of the invitation pack.

Alternatively, on receipt of the patient’s Consent to Contact form, a member of the MDT will contact the patient and complete the first section of the Screening Form over the telephone. If potentially eligible, the patient will be invited to make an appointment in clinic for a face-to-face screening to check eligibility and complete the outstanding screening questions. If eligible, patients will follow the consent process.

Some patients may not meet all of the inclusion criteria at the initial screening and would be eligible for a rescreen for two reasons: patient has not yet had a stable residual limb for at least 3 months, or they presently have a contraindication for wearing their current prosthesis. They will be contacted after 3 months to reassess their eligibility unless they explicitly request otherwise. Patients will only be rescreened for eligibility once.

### Study visits and follow-up

Data will be collected from participants at four time points.

#### Screening visit

Initial screening may be conducted by telephone, but all prospective participants must have a final face-to-face screening at their prosthetics centre by a member of their normal MDT for eligibility. Informed consent will be sought from eligible patients by an MDT member and according to Good Clinical Practice guidelines.

#### Baseline assessment

All consenting participants will be asked to complete a baseline questionnaire and undergo four clinical assessments at the prosthetics centre. They will be loaned an activity monitor (activPAL4, PAL Technologies Ltd, Glasgow, UK), which will be fitted onto their prosthesis for 1 week after the baseline assessment. After this, they will be requested to remove it and return it via post. The activity monitor will quantify the time spent during walking activities and the number of daily steps taken, using their standard prosthesis.

#### Interim follow-up

All participants will be posted a questionnaire pack mid-way through the intervention period (week 9 post-randomisation). They will be asked to complete and return the questionnaires in a prepaid return envelope.

#### Final follow-up

Participants will be posted an activity monitor in the week prior to their final follow-up assessment (15 weeks post-randomisation), and asked to fit the activity monitor onto their prosthesis to monitor their daily stepping with the prosthesis they are currently using. The activity monitor will be removed when the participant attends the prosthetics centre for their final study visit. Participants will also be asked to complete the same questionnaire and clinical assessments as they did at baseline.

### Outcome measures

Table 1 details the information to be collected according to time points. The participant questionnaires include the following.

- Locomotor Capabilities Index-5.^20^

**Table 1:** Trial activity and data collection time points

	Screening	Baseline assessment (in clinic)	Prosthetic fitting (weeks 1–3)	Week 9 follow-up assessment (postal)	Week 15 assessment (postal)	Week 16 follow-up assessment (in clinic)
Eligibility screening form	**X**					
Consent		**X**				
Questionnaires						
Demographics		**X**				
LCI-5		**X**		**X**		**X**
Houghton		**X**		**X**		**X**
EQ-5D-5L		**X**		**X**		**X**
PROMIS 3a ānd 8a		**X**		**X**		**X**
Healthcare resource use		**X**		**X**		**X**
Clinical assessments						
2mWT		**X**				**X**
TUG		**X**				**X**
TUDS		**X**				**X**
BBS		**X**				**X**
1 week activity monitor		**X**			**X**	
Novel prosthesis treatment group only			**X**			

BBS, Berg Balance Scale; LCI-5, Locomotor Capabilities Index-5; PROMIS, Patient-Reported Outcomes Measurement Information System; TUDS, Timed Up and Down Stair test; TUG, Timed Up and Go test; 2mWT, 2 min walk test.

- Houghton Scale—prosthetic use in people with lower extremity amputations.^21^
- Patient-Reported Outcomes Measurement Information System (PROMIS) Short-Form v1.0 Pain (3a and 8a) questionnaires.^22^
- EQ-5D-5L.^23^
- Bespoke health resource use questionnaire.

The four clinical assessments will be carried out at baseline with the participant wearing their standard prosthesis and repeated at final follow-up, wearing whichever prosthesis they are using at the time. Participants may also use their regular walking aid and choose not to attempt some assessments. The assessments are as follows.

- 2-min walk test.^24^
- Timed-Up and Go test.^25^
- Timed-Up and Down test.^26^
- Berg Balance Scale.^27^

### Qualitative data collection

Semi-structured interviews will be undertaken with 20–25 participants to explore their experience of being involved in the trial, acceptability of the study design, randomisation and other study processes, and acceptability of the intervention. Participants will be purposively selected, we aim to sample approximately 8–10 participants from each of the novel prosthesis and standard prosthesis arms, and approximately five patients who declined to participate or dropped out of the trial to explore reasons for non-participation. We appreciate that the non-participant group may be difficult to reach and will only approach individuals who, at the time they declined to participate, indicated they were happy to be contacted with a view to being potentially interviewed. Purposive selection will ensure maximum variation across the sample with regard to age and gender. The proposed sample of interviewees is likely to achieve data saturation whereby similar themes emerge.^28 29^ A flexible interview schedule will be developed.

Focus groups will also be conducted with staff (including clinicians, physiotherapists, prosthetists and centre managers) at each participating prosthetics centre to discuss any barriers to successful delivery of the trial. Findings from the qualitative work will be integrated with the feasibility trial outcomes in order to inform the design of a full-scale RCT.

All interviews and focus groups will be audio recorded with permission. Recordings will be transcribed verbatim by approved transcribers and anonymised. A second researcher will check a sample of data transcripts against the audio recordings for accuracy, and will interrogate the validity of the coding against the raw data. Data will be entered into appropriate software for thematic analysis.

### Statistical analyses

A statistical analysis plan detailing intended analyses will be drafted before the completion of data collection. The trial will be reported according to the CONSORT guidelines for feasibility and pilot trials and the flow of participants through the trial will be detailed in a CONSORT flow diagram.

Baseline data will be summarised by trial arm, as randomised, with no formal comparison between the groups. Continuous data will be reported descriptively (mean, SD, median, minimum, maximum and number missing), and categorical data by counts and percentages. No formal statistical analyses will be undertaken as this is a feasibility study. Completion rates of all the clinical outcome measures will be reported by trial arm and overall.

The recruitment rate will be reported monthly, and overall, by centre. An average monthly recruitment rate will be calculated, and a 95% CI will be estimated from the data collected. This could be used to inform recruitment rates per site in a future main trial and determine if an adequate sample could be recruited in ≥24 months in a larger trial. The number of eligible patients will be summarised overall, by site, using counts and percentages. Reasons for ineligibility will be detailed in the CONSORT diagram. The following will also be reported, the proportion of:

- Eligible patients approached for consent.
- Patients approached who provide consent.
- Patients approached who do not provide consent.
- Patients providing consent who are randomised.
- Patients dropping out between randomisation and follow-up.

### Health economic analysis

A full cost-effectiveness analysis will not be undertaken as part of the current study. Rather, the work will identify the feasibility of collecting the data, needed for an economic analysis of a full-scale trial. Health service resource use will be collected from participants using a bespoke questionnaire that will include items such as hospital attendances and admissions and also primary care visits (eg, GP, physiotherapist and prosthetist). The costing approach will be undertaken from an NHS perspective and unit costs will be derived from established national costing sources such as NHS Reference Costs^30^ and Personal Social Services Research Unit costs of health and social care.^31^ The costs of providing the novel prosthesis will be estimated and the potential resource implications versus the standard prosthesis will be explored.

### Safety measurements

Adverse events (AEs) related to the prosthesis only, and any serious adverse event (SAE), will be recorded throughout the study. Intensity and relationship to the study intervention will be described. Ongoing review of AEs and SAEs will take place during monthly Trial Management Group (TMG) meetings, discussed with the Patient Advisory Group (PAG) and Trial Steering Committee (TSC), and reported to the sponsor and ethics committee in line with their guidelines. Participants may withdraw from the study at any time without influencing their future care or treatment.

### Trial monitoring and oversight

Due to the low risk nature of this trial, there will be one independent steering and monitoring committee to undertake the roles traditionally undertaken by the TSC and Data Monitoring and Ethics Committee. This Trial Steering and Monitoring Committee will comprise of independent members including a Chair, a statistician, one other independent person, and a member of the PAG.

The TMG, comprising the chief investigator, the York Trials Unit and coinvestigators, will provide overall management of the study. York Trials Unit is responsible for project management. We will establish a PAG with between four and eight members that will meet a minimum of five times over the duration of the project. The PAG is a group of independent patients with an amputation and their carers, whose role is to support and advise the TMG on all aspects of the study and to facilitate its progress and management.

### Patient and public involvement

Three funded patient and public involvement (PPI) events were carried out at the recruiting sites to inform the development of this study. The PPI events helped to identify the objectives for the feasibility study, specify outcomes that are significant to this patient population, and share views on the randomisation process. This has enabled the team to prioritise outcome measures related to amputee patient function and well-being, and explore how to introduce the study to potential participants while taking into account their valid concerns about randomisation.

### Data collection, integrity and management

Data will be collected through paper questionnaires identified by a unique identification number only (ie, the participant identification number in all manual and electronic files). Each site will hold data according to the General Data Protection Regulation (May 2018). A Trial Enrolment Log at the sites will list the participant identification numbers. York Trials Unit will maintain a list of participant identification numbers for all trial patients at each site.

All paper documents will be stored securely. All information collected will be stored on a secure password-protected server located at the University of York, for the purpose of assisting in follow-ups during the study and will be kept strictly confidential.

The confidentiality of participants and staff interviewed during the qualitative interviews will be ensured by assigning a unique participant number to electronic sound files and transcripts, known only to the qualitative researcher and appropriate members of the research team. Any quotes published will be anonymous. All data collected will be archived for ten years following the end of the study.

### Ethics and dissemination

Yorkshire and The Humber—Leeds West Research Ethics Committee granted ethical approval for this study on 4 May 2018. Since the study started, the Health Research Authority approved three substantial amendments to the protocol which are included in the final version reported here.

1. Inclusion of clear written and pictorial instructions for fitting the activity monitor to the participant’s prosthesis.
2. Alteration to the recruitment process such that staff at the local prosthetics centre could contact a potential participant after invitations packs had been sent to them.
3. Inclusion of a cover letter to accompany the participant information sheet for the qualitative interview aspect of the study.

The proposed study will be conducted in accordance with the Medical Research Council Guidelines on Good Clinical Practice in Clinical Trials.

The results will inform the design and delivery of a definitive RCT. The findings from this study will be presented to relevant groups such as the British Association of Chartered Physiotherapists in Amputee Rehabilitation (BACPAR) and the British Association of Orthotists and Prosthetists (BAPO). Participants will be informed of the outcome of the study. With the help of our PAG representatives, the findings will be disseminated to participants, other patients and their carers, and relevant patient support groups, including national charities supporting individuals following limb loss. The results will also be submitted to the funders, peer-reviewed journals, presented at relevant meeting/conferences, including participating recruitment sites.

## Conclusion

There is limited robust evidence about the benefits of prescribing a self-aligning prosthesis for patients over the age of 50 years, who are predominantly prescribed a standard (ie, more rigid) prosthesis. The outcomes from this study will inform a larger fully powered RCT designed to determine the effectiveness of a self-aligning prosthesis in improving a patient’s daily mobility.

## Supplementary Material

Reviewer comments
