# Supplementary material for: Self-aligning prosthetic device for older patients with vascular-related amputations: protocol for a randomised feasibility study (the STEPFORWARD study)
Source: BMJ Open. 2019 Sep 20;9(9):e032924. doi: 10.1136/bmjopen-2019-032924 (PMC6756612; doi:10.1136/bmjopen-2019-032924)
Supplement: Reviewer comments [file bmjopen-2019-032924.reviewer_comments.pdf]

PEER REVIEW HISTORY

BMJ Open publishes all reviews undertaken for accepted manuscripts. Reviewers are asked to complete a checklist review form (**http://bmjopen.bmj.com/site/about/resources/checklist.pdf**) and are provided with free text boxes to elaborate on their assessment. These free text comments are reproduced below.

ARTICLE DETAILS

**TITLE (PROVISIONAL)**	A self-aligning prosthetic device for older patients with vascular-related amputations: Protocol for a randomised feasibility study (the STEPFORWARD study)
**AUTHORS**	Vanicek, Natalie; Mitchell, Natasha; Coleman, Elizabeth; Watson, Judith; Bell, Kerry; McDaid, Catriona; Barnett, Cleveland; Twiste, Martin; Jepson, Fergus; Salawu, Abayomi; Harrison, Dennis

This article was not externally reviewed at BMJ Open. Protocols that have been independently assessed prior to submission to BMJ Open are usually fast-tracked to publication on the grounds that further substantial changes will not be possible. This independent assessment will usually be external, independent review for both a substantial grant award from a non-commercial or government funder and ethics approval.
